# Endocytosis and Transcytosis of SARS-CoV-2 Across the Intestinal Epithelium and Other Tissue Barriers

**DOI:** 10.3389/fimmu.2021.636966

**Published:** 2021-09-07

**Authors:** Evgeny Knyazev, Stepan Nersisyan, Alexander Tonevitsky

**Affiliations:** ^1^Laboratory of Microfluidic Technologies for Biomedicine, Shemyakin-Ovchinnikov Institute of Bioorganic Chemistry of the Russian Academy of Sciences, Moscow, Russia; ^2^Faculty of Biology and Biotechnology, National Research University Higher School of Economics (HSE), Moscow, Russia

**Keywords:** SARS-CoV-2, COVID-19, coronavirus, intestines, gastrointestinal tract, microfluidics, transcytosis, endocytosis

## Abstract

Since 2003, the world has been confronted with three new betacoronaviruses that cause human respiratory infections: SARS-CoV, which causes severe acute respiratory syndrome (SARS), MERS-CoV, which causes Middle East respiratory syndrome (MERS), and SARS-CoV-2, which causes Coronavirus Disease 2019 (COVID-19). The mechanisms of coronavirus transmission and dissemination in the human body determine the diagnostic and therapeutic strategies. An important problem is the possibility that viral particles overcome tissue barriers such as the intestine, respiratory tract, blood-brain barrier, and placenta. In this work, we will 1) consider the issue of endocytosis and the possibility of transcytosis and paracellular trafficking of coronaviruses across tissue barriers with an emphasis on the intestinal epithelium; 2) discuss the possibility of antibody-mediated transcytosis of opsonized viruses due to complexes of immunoglobulins with their receptors; 3) assess the possibility of the virus transfer into extracellular vesicles during intracellular transport; and 4) describe the clinical significance of these processes. Models of the intestinal epithelium and other barrier tissues for *in vitro* transcytosis studies will also be briefly characterized.

## Introduction

At the moment, it remains unclear whether the gastrointestinal tract can serve as an entry portal for SARS-CoV-2; however, fecal-oral transmission is considered likely (1). There is a concern that SARS-CoV-2 transmission may occur *via* frozen foods or packaging (2), so the fate of the coronavirus in the intestine is relevant. SARS-CoV-2 RNA was detected in feces of infected people in >50% of cases, and >20% of patients continued to have positive stool results after negative respiratory sample results (3). The SARS-CoV-2 nucleocapsid (N) protein was found in the cytoplasm of gastric, duodenal, and rectal epithelial cells. Viral RNA was also detected in samples of the esophageal mucosa, although the N-protein was not detected in esophageal epithelial cells (3). A systematic review and meta-analysis of 35 studies showed that ~15% of patients had digestive symptoms including nausea, vomiting, and diarrhea, while 10% of patients had gastrointestinal symptoms alone without respiratory features (4). According to electron microscopy and virus culture data, active viral replication of SARS-CoV-2 was detected in both the small and large intestines (5). SARS-CoV-2 cell entry depends on angiotensin-converting enzyme 2 (ACE2) and transmembrane serine protease 2 (TMPRSS2) (6), which are both expressed on the luminal surface of cells in the gastrointestinal tract (3, 7). SARS-CoV-2 can cause productive infection of human enterocytes (8). These facts support the possibility of fecal-oral transmission of SARS-CoV-2, and underscore the need to elucidate possible mechanisms of coronavirus penetration through tissue barriers, including in the intestine.

## Native and Opsonized Coronaviruses

To understand and model the processes of endocytosis and transcytosis of SARS-CoV-2 in the intestine and other barrier organs, it is necessary to have a clear understanding of the structure of native virions, including their size, the number of cell-interacting spikes on the virion surface, the mechanism and stoichiometric ratios in the interaction of spines with receptors and antibodies, and corresponding structural limitations.

A combination of cryoelectron tomography and subtomogram averaging for a study of ~2300 intact SARS-CoV-2 virions showed ellipsoidal envelope particles with average diameters of 64.8 ± 11.8, 85.9 ± 9.4, and 96.6 ± 11.8 nm (average ± SD) for the short, medium, and long axes of the envelope, respectively (9). About 97% of spike (S) protein trimers in this virion sample were in prefusion conformation and 3% in postfusion, with each virion having an average of 26 ± 15 spikes in prefusion conformation and each spike freely changing the angle relative to the virion membrane (9). In another study, the average virion diameter, if taken as spherical, was 91 ± 11 nm, and each virion had 24 ± 9 S-protein trimers, 3% of which were in postfusion conformation. A small sub-population of virions contained only few S trimers, while larger virions contained more S trimers. There was approximately one spike per 1,000 nm^2^ of virus membrane (10). For comparison, there were approximately 10 spikes of the influenza A virus per 1,000 nm^2^ of membrane surface with a virion diameter of 80–120 nm (11). That is, the distances between the spikes of the influenza A and SARS-CoV-2 are approximately 11 nm and 35 nm, respectively. In another study, <0.1% of SARS-CoV-2 S-protein trimers were in postfusion conformation, which can be explained by the type of cells in which virions were produced, including the number of receptors and proteases involved in the transition of receptors into postfusion conformation (12). As it was shown, each virion had an average of 40 spikes evenly distributed over the surface without a tendency of agglomeration (12). Each trimer freely bent at an angle to the membrane due to a stalk with three flexible hinges. In contrast to recombinant S-proteins, trimers synthesized in infected cells were much more densely covered with glycans, mainly due to N-glycosylation (12).

The free arrangement and rotation of S-protein trimers apparently helps the virus to “explore” the surrounding space, allowing several spikes to interact with one ACE2 receptor or one spike with several ACE2 molecules simultaneously (9). The ACE2 homodimer can interact with two S-proteins of two different spikes, in which one S protein is in an up conformation, and the other two are in down conformations (13). D. Ni and colleagues (14) published work indicating possibility of the simultaneous interaction of three free ACE2 ectodomains with the SARS-CoV-2 trimeric spike, which led to the transition of all three S-proteins into up conformation and disassembly of the trimeric form. Although the conclusions of this investigation must be peer-reviewed, preliminary conclusions can be drawn about the possibility of the S-protein trimer interacting with more than one ACE2 molecule. In another study, SARS-CoV-2 S-protein trimers were mixed with ACE2 molecules in the presence or absence of dp20 oligosaccharides derived from heparin. After incubation for 60 minutes, transmission electron microscopy revealed that in the absence of dp20 oligosaccharides, the proportions of S-protein trimers bound to one, two, and three ACE2 molecules were 45%, 11%, and 13%, respectively, and in the presence of dp20 oligosaccharides the corresponding values were 40%, 19%, and 27%. The authors concluded that each SARS-CoV-2 spike can interact with more than one ACE2 molecule, and the presence of heparin can stabilize this interaction (15).

The sparse arrangement and free rotation of SARS-CoV-2 spikes also makes them more vulnerable to neutralizing antibodies, which provides an additional opportunity to bind to hard-to-reach domains (16) and glycan holes of the S protein (17). An immunoglobulin G (IgG) molecule can potentially bind both to one coronavirus spike and two adjacent spikes due to two Fab domains of IgG (18). Cryoelectron microscopy has also shown that one trimer of the SARS-CoV-2 S protein can bind to either one or two or three Fab domains (19, 20), potentially indicating the ability to bind up to three antibodies to one SARS-CoV-2 spike. Another study showed the bivalent interaction of full-length IgG with a trimer of the SARS-CoV-2 S-protein (21). Given the linear dimensions of antibodies (22), it can be assumed that an IgG-coated SARS-CoV-2 virion will have a diameter no more than 20 nm larger than the free virion. When interacting with polymeric immunoglobulin A (IgA) and M (IgM), the size may increase even more. Ig molecules can also bind to spikes of neighboring virions, leading to the formation of agglomerates.

Thus, the coronavirus can interact with target cells both in the form of a free virion and in the form of an opsonized virion or viral agglomerates, cross-linked by antibodies. In each case, the particle size and molecules available on their surface can determine various mechanisms of endocytosis and subsequent intracellular transport. Possible mechanisms of coronavirus binding to target cells are briefly discussed below.

## How Coronaviruses Bind Cells

The process of virus penetration into the body through barrier tissues begins with cell binding. The fate of the virus may depend on many factors including the shape and structure of the virion, the structure and number of cell receptors and the corresponding virus-binding molecules, the possibility of dimerization/polymerization of these receptors and molecules, the structure of the membrane surrounding the receptors (e.g., lipid rafts, etc.). Viruses can penetrate and infect cells of barrier tissues directly through the cytoplasmic membrane and indirectly by using cell endocytosis pathways. A number of viruses can also be internalized by the cell and cross the tissue barrier due to transcytosis (23, 24). There are several distinct stages in the process of virus interaction with a cell, including attachment to the cell surface, lateral movement along the cytoplasmic membrane and receptor clustering, activation of cellular signaling pathways, endocytosis and transport to secondary organelles, cell entry by fusion, lysis or pore/channel formation, intracellular transport to the nucleus or compartments within the cytoplasm, and complete or partial uncoating of viral particles (24).

Since viral particles can simultaneously bind several molecules on the cell surface, virions are more likely to bind to microdomains of the cell membrane containing receptor clusters, leading to further clustering of receptors or rearrangement of these microdomains (25). We noted above that SARS-CoV-2 has a high degree of spike mobility, and each one can bind to several molecules on the cell surface, which facilitates receptor clustering and virus cell entry. A ganglioside-binding domain was predicted in the N-terminal domain of the SARS-CoV-2 S-protein, which may be responsible for the binding of coronavirus spikes to lipid rafts rich in gangliosides and sialic acids (26). Molecular mimicry was also observed between azithromycin and ganglioside sugars, and it was suggested that the decrease in viral load following azithromycin treatment for COVID-19 can be explained by the fact that azithromycin binds SARS-CoV-2 S-proteins, preventing their interaction with lipid rafts (27). Other coronaviruses have also shown tropism for membrane microdomains. For MERS-CoV, tetraspanin CD9 in the cell membrane microdomains promotes the formation of complexes of the MERS-CoV receptor dipeptidyl peptidase 4 (DPP4) and the TMPRSS2 molecule with effective early cell entry of the virus; in the absence of this tetraspanin, MERS-CoV was not activated by membrane serine proteases, so it was transported to endosomes and activated by cathepsins (28). For SARS-CoV, ACE2 receptor concentration in cholesterol-rich lipid rafts is important for virus cell entry. Destroying lipid rafts while maintaining ACE2 expression significantly reduced the virus ability to enter the cell (29, 30). Since the cell entry mechanisms of SARS-CoV-2 and SARS-CoV are similar, it can be assumed that cytoplasmic membrane microdomains also play an important role in the interaction between cells and SARS-CoV-2.

Intracellular viral fate may depend on whether a free virion or an opsonized viral particle attaches to the cell surface, since distinct receptors are involved in virus attachment for these scenarios. For adenoviruses and rhinoviruses, it is assumed that aggregates of virions enter the cell in a different way than individual viral particles (31, 32), which may also be important for coronaviruses. The following section discusses potential mechanisms of coronaviruses bind to cells of barrier.

### Binding of Native Virions Through the Interaction of S-Proteins and Specific Target Cell Receptor Molecules

For SARS-CoV, SARS-CoV-2, and MERS-CoV, mechanisms of cell entry by interacting with specific target molecules are known: ACE2 for SARS-CoV and SARS-CoV-2 and DPP4 for MERS-CoV (33). S-proteins of coronaviruses are typical class I fusion proteins that require prefusion cleavage with proteases such as furin, cathepsins, TMPRSS2 and TMPRSS4, human airway trypsin-like protease (HAT), trypsin, thermolysin, and elastase, depending on the virus and cell type (34, 35). S-protein cleavage by proteases is necessary to initiate fusion of the virus and cell membranes, which occurs during endocytosis. Interestingly, cleavage of the ACE2 receptor molecule was also noted, followed by SARS-CoV virus endocytosis (36) and the further possibility of transcytosis or cytoplasmic penetration through late endolysosomes/lysosomes.

There are recent reports that neuropilin-1 (NRP1) protein can serve as another SARS-CoV-2 receptor (37, 38). This protein interacts with furin-cleaved substrates, and the interaction of furin-cleaved SARS-CoV-2 S protein and NRP1 was confirmed using crystallographic and biochemical approaches. Inhibition of this interaction by RNA interference or selective inhibitors reduced SARS-CoV-2 infectivity *in vitro* (38). Conversely, NRP1 overexpression enhances SARS-CoV-2 infectivity *in vitro*, and monoclonal antibodies to NRP1 counteract this effect. A mutation in the furin cleavage site of the SARS-CoV-2 S-protein resulted in loss of the dependence of virus infectivity on NRP1 level. Interestingly, olfactory neuronal cells highly express NRP1, which may explain the COVID-19 symptom of anosmia (37). These data indicate that beyond ACE2, SARS-CoV-2 may have one more specific receptor on the surface of target cells. In addition to the specific mechanism of viral penetration into the cell through interaction of a receptor molecule with coronavirus spikes, there is also the possibility of absorption of opsonized viral particles due to the interaction of the fragment crystallizable (Fc) region of antibodies with Fc receptors on the target cell surface.

### Binding of Opsonized Virions Through Interactions Between Immunoglobulins and Their Receptors

Normally, opsonized virion absorption with aid of the Fc receptors is aimed at neutralizing viral particles by immune cells; however, there is a phenomenon of antibody-dependent enhancement (ADE) that occurs when a low level of antibodies or their low neutralizing activity can contribute to cellular infection with opsonized viruses (39). Fc receptors are also present on the surface of epithelial cells of the gastrointestinal tract, respiratory system, kidneys, liver, and placental trophoblast (40), which also raises the possibility of antibody-mediated penetration of coronaviruses into these cells and transcytosis through tissue barriers. Detailed investigation of the mechanism of SARS-CoV-2 neutralization with antibodies is a very urgent task since it underlies the action of vaccines and the use of blood plasma of people who recovered from COVID-19, immunoglobulins obtained from this plasma, and recombinant antibodies and their fragments directed against viral proteins (41, 42). In this case, the most important parameters are the titer and antigenic specificity of neutralizing antibodies necessary to suppress a viral infection (41). When choosing the antibody concentration, it is necessary to consider the possibility of antibody-mediated penetration of coronaviruses through tissue barriers or development of ADE. Viral protein immunogenicity, epitope mapping, the affinity of various antibodies, and the variability of the amino acid sequence in the region of a number of epitopes are being studied in detail; these factors can weaken the specificity of the antibodies produced and, as a consequence, weaken antiviral immunity (43–45). Recently published results indicate the importance and relevance of these studies (46).

IgA molecules predominate the secretions of most mucous membranes including the intestinal mucosa; these are synthesized by immune cells in the lamina propria, after which the polymeric immunoglobulin receptor (pIgR) binds IgA dimers on the basolateral membrane of epithelial cells and promotes IgA transcytosis towards the apical membranes. In a similar way, pIgR carries out transcytosis of IgM pentamers, the function of which is like IgA, but the relative amount in mucous membrane secretions is significantly less. On the apical membrane surface or intracellularly during transcytosis, pIgR undergoes proteolytic cleavage, and its extracellular domain in complex with IgA or IgM is separated from the membrane (47). However, one group reported that the cleavage half-time of pIgR on the surface of the apical membrane is 5–10 min, so intact pIgR molecules remain on the membrane (48). In polarized MDCK cells transfected with human pIgR, endocytosis of the pIgR ligand together with the receptor on the apical membrane was possible; however, this complex predominantly recirculated back to the apical membrane or remained inside the cell, and only 5% of the internalized pIgR complexes with the ligand entered the basolateral compartment over 90 min (48). Probably, due to the low level of pIgR transcytosis in MDCK model, this receptor should not play a significant role in the penetration of SARS-CoV-2 through the cell barriers. It remains unclear whether the direction of IgA and IgM transport in polarized cells can be changed during SARS-CoV-2 infection. It is also unclear whether IgA and IgM complexes with antigens significantly contribute to coronavirus penetration through epithelial barriers by increasing transport from the apical to basolateral membrane. In Peyer’s patches in the intestinal epithelium, there are so-called M-cells that are able to bind secretory IgA (sIgA) in combination with antigens and transport them towards the basolateral membrane for immune cell presentation, but the sIgA receptor in these cells remains undefined (49). At the same time, M-cells remain a possible gateway for SARS-CoV-2 to penetrate the intestinal epithelial barrier.

Another class of antibodies present in mucous membranes secretions is IgG. In lower respiratory tract and genitourinary system secretions, IgG predominates over IgA, and in the mucous membrane of the gastrointestinal tract, upper respiratory tract, and other areas, IgG levels can significantly increase in response to infection (47). Recognition of IgG Fc regions can occur with the involvement of FcγR and FcRn receptors. FcγR family receptors include three classes (I, II, and III); however, they are predominantly expressed on immune cells (50) and therefore do not play a special role in IgG transcytosis across epithelial barriers.

The FcRn molecule is a heterodimer of two subunits linked by non-covalent bonds. The p51 subunit, or 40-kDa heavy α-chain, is encoded by the *FCGRT* gene and directly interacts with the Fc region of IgG; the 12-kDa p14 subunit is a β2-microglobulin encoded by the *B2M* gene. The heavy α-chain has three extracellular domains (α1, α2, and α3), a transmembrane domain, and a cytoplasmic tail of 44 amino acids (40). FcRn can bind with high affinity to the Fc region of IgG in weakly acidic conditions (pH 5.0–6.5); at neutral pH, weak binding was found only to IgG3 allotypes (51). At the same time, IgG3 appears at the earliest stages of viral infection and has the most pronounced effector activity, which contributes to the rapid development of a powerful immune response during infection, but it can lead to hyperreactivity and stimulate immune-mediated damage to one’s own tissues (52). IgG3 also contributes to ADE of infection with the enveloped Dengue virus and Ebola virus (52).

According to the methodology (53) we have analyzed expression profiles of *FCGRT* and *B2M* in human tissues from 11 different organs based on RNA sequencing within the framework of The Cancer Genome Atlas project. We have also analyzed the gene expression levels of *NRP1* and *NRP2*, which were shown to interact with furin-cleaved S-proteins, and *ACE2* and *TMPRSS2*, which are considered the main participants in SARS-CoV-2 cell entry. High expression levels of *FCGRT* and *B2M* were observed in the gastrointestinal tract organs and lungs, and expression levels were significantly higher than for *NRP1*, *NRP2*, *ACE2*, and *TMPRSS2*. This may indicate the possibility of antibody-dependent penetration of coronaviruses through organ barrier tissues. High *FCGRT* and *B2M* expression were also noted in the liver, which may indicate a potential mechanism of the liver damage observed in a number of COVID-19 patients (54).

## Coronavirus Endocytosis

After binding to the cell surface, viruses can be endocytosed into the cell. The endocytosis of quantum dots coated with the SARS-CoV-2 S-protein in complex with ACE2 molecules with attached C-terminal green fluorescent protein was monitored in HEK293T cells using inclined/total internal reflection fluorescence illumination microscopy. Binding of quantum dots to ACE2 receptors on the cell surface and endocytosis into cells was detected within minutes, but this was suppressed by a dynamin inhibitor and neutralizing antibodies to ACE2, which may indicate a clathrin-mediated endocytosis mechanism (55). Lentivirus pseudotyped with SARS-CoV-2 S-protein engaged with the plasma membrane and entered the cell by clathrin-mediated endocytosis, and knockdown of clathrin heavy chain reduced viral infectivity (56).

The cytoplasmic tail of the ACE2 molecule contains a class I PSD-95/Dlg/ZO-1 (PDZ) binding motif, an endocytic motif for the clathrin-adaptor subunit AP2 μ2, and an LC3-interacting region motif (57). The class I PDZ binding motif in the ACE2 cytoplasmic tail binds the first PDZ-domain of the scaffold protein NHERF3, and the clathrin-adaptor subunit AP2 µ2 interacts with an endocytic motif in the ACE2 with low affinity; this interaction is abolished by Tyr781 phosphorylation (58), which indicates a potential mechanism of involvement in clathrin-mediated endocytosis. The LC3-interacting region motif mediates the interaction with the ATG8 domain containing microtubule associated protein 1 light chain 3 (MAP1LC3) proteins and GABA type A receptor-associated protein (GABARAP) in the phagophore membrane (59), suggesting the possibility of autophagy mechanisms following ACE2-dependent endocytosis. It was previously shown that upon binding to ACE2, SARS-CoV also enters the cell *via* clathrin-mediated endocytosis; however, the degree of clathrin-mediated endocytosis was not affected in cells expressing modified ACE2 without a cytoplasmic tail (60). The possibility of clathrin- and caveolin-independent endocytosis of SARS-CoV after ACE2 interaction was also demonstrated (61). Collectively, it indicates the need for additional research to clarify the mechanisms of endocytosis of coronaviruses after interaction with ACE2.

As was mentioned above, NRP1 can also serve as a SARS-CoV-2 receptor (37, 38), which suggests additional endocytosis mechanisms. NRP1 binds molecules with a C-terminal, basic sequence motif (C-end Rule or CendR motif). It was shown that CendR-associated endocytosis is not clathrin- and caveolae-mediated (62). Moreover, ultrastructurally this type of endocytosis resembles macropinocytosis but is mechanistically different. Specifically, it is receptor-associated and not inhibited by Cdc42 or Rac1 knockdown and weakly decreases when the macropinocytosis inhibitor rottlerin is used (62). NRP1 contains a PDZ binding motif that interacts with the PDZ-domain of the cytoplasmic protein GIPC1/synectin to play a role in trafficking endocytosed VEGFR2 into Rab5a-positive endosomes (63). NRP1-GIPC1/synectin interaction is not needed for CendR peptide binding, but it is important for cellular internalization and is enhanced by rapamycin treatment and depleting glucose or amino acids in the culture media through inhibition of the mammalian target of rapamycin pathway (62). However, during NRP1-associated endocytosis cargo ends up in endosomes and multivesicular bodies (62); therefore, despite the type of virus endocytosis, potential pathways for further intracellular sorting may intersect.

In addition to the specific mechanism of cell entry *via* a receptor molecule, there is also the possibility of endocytosis of opsonized viral particles that occurs due to interaction of Fc regions of antibodies on the surface of the virus with Fc receptors of target cells. Since Fc receptors are highly expressed on immune cells, a large number of studies have been devoted to the study of ADE in the context of immune cell infection. Passive immunization of cats with antibodies to the feline infectious peritonitis virus belonging to the coronavirus family can lead to ADE, and *in vitro* antibodies to this virus cause antibody-dependent infection of macrophages while maintaining virus activity (64). Antibodies to SARS-CoV promoted the infection of monocytes and B-line lymphoblasts mediated by FcγR and was independent of ACE2 receptor expression, pH level, and cysteine protease activity (65). Antibodies to SARS-CoV led to viral penetration into human macrophages with the participation of FcγR—in particular FcγRIIA and FcγRIIB—with the synthesis of a structural viral N-protein; however, the virus did not replicate productively, and the immune cells did not release new viral particles (66). When diluted 10 times, the anti-sera of patients recovered from SARS-CoV infection had an *in vitro* antiviral effect when infecting the monocytic cell line HL-CZ, but dilution of the anti-sera by 100, 1000, and 2000 times increasingly enhanced infection (67). In addition, during the development of antibodies to S- and N-proteins SARS-CoV in mice, infection enhancement was achieved due to antibodies to S protein. The authors found that mouse anti-spike protein serum significantly reduced HL-CZ cell infection when diluted 10 times and markedly enhanced infection when diluted 1000 and 2000 times (67).

The S230 antibody to the receptor-binding domain of SARS-CoV S protein prevented interaction of the virus with ACE2 but stimulated transition of the S protein to the postfusion conformation, mimicking the interaction with ACE2, while the LCA60 antibody to the side of the receptor-binding domain of MERS-CoV S protein prevented interaction with DPP4 and stabilized the prefusion conformation (17). Interaction of MERS-CoV S-protein with antibodies to the receptor-binding domain can facilitate viral particle fusion with cells, mimicking the interaction with DPP4 (39). Antibodies to a specific epitope of the SARS-CoV S-protein caused ADE both *in vitro* in Vero E6 cells and *in vivo* in monkeys (68). Vaccination of monkeys with a modified vaccinia Ankara virus encoding full-length SARS-CoV spike glycoprotein and their subsequent infection with SARS-CoV resulted in a significantly lower viral load after vaccination. However, IgG-associated acute lung injury developed due to pro-inflammatory macrophage activation, which decreased upon FcγR blockade (69). Apparently, a suboptimal immune response can lead to ADE and cytokine release syndrome, aggravating the course of infection (70–73). Antibodies to coronaviruses can stimulate viral particle endocytosis into cells expressing Fc receptors. The ultimate fate of the virus in the cell is determined both by the type of receptor and intracellular transport pathway, as well as the antigenic specificity and affinity of the bound antibody. Taken together, these factors determine the possibility of fusion of the viral and intracellular organelle membranes or exocytosis and transcytosis of the viral particle.

## Coronavirus Transcytosis

Transcytosis is the transport of macromolecules, supramolecular complexes, and even microorganisms through the cell using membrane-bounded carriers. As a rule, the process is characteristic of polarized cells that form tissue barriers between two environments. Transcytosis can be carried out using various mechanisms; for example, one of the variants can be considered as a branch of endocytosis when the transported molecules are internalized using receptor-dependent mechanisms. This occurs most often through clathrin-coated vesicles; they enter the sorting membrane compartments of cells and then are released from cells by exocytosis, which is more typical for intestinal cells. Another variant, more typical for endothelial cells, is dependent on caveolae and a more direct route of transcytosis without entering intermediate compartments (74). It seems that viruses can use both types of transcytosis, which will be discussed later in this review.

Virus transcytosis through polarized cells can begin both with endocytosis upon binding to a specific target molecule and with macropinocytosis or phagocytosis. The main transcytosis pathway through the vascular endothelium of the lungs is predominantly caveolae dependent (75), and equine encephalitis viruses also penetrate the blood-brain barrier *via* caveolae-mediated transcytosis (76). Human immunodeficiency virus (HIV) can penetrate through the epithelial barriers in models of the endometrium (cell line HEC-1) and intestine (cell lines Caco-2, HT-29 and I407) by transcytosis without infecting the cells, but the presence of neutralizing sIgA and IgG disrupts transcytosis (77). Epstein-Barr virus from the herpesvirus family penetrates the polarized epithelium of the oral cavity using transcytosis, while an inhibitor of micropinocytosis (amiloride) reduced the degree of transcytosis (78). Caco-2 cells cultured in the presence of human lymphocytes from Peyer’s patches acquired the M-cell phenotype and became capable of transcytosis from the apical to the basolateral membrane, even for large microorganisms such as *Vibrio cholerae* (79).

**Figure 1:**
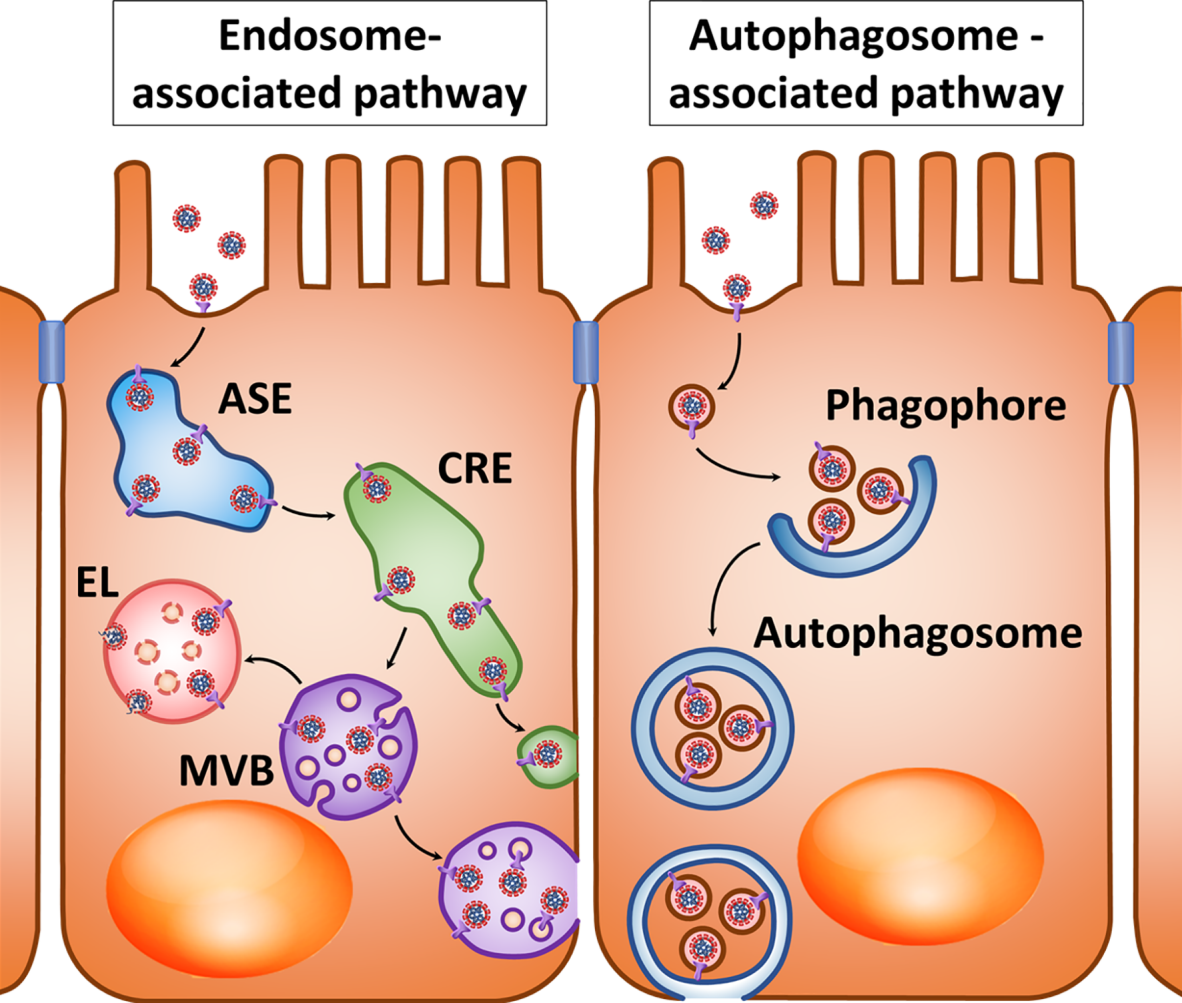
Hypothetical pathways for SARS-CoV-2 transcytosis after interaction with ACE2. *Left*: After interacting with ACE2, virions enter the apical sorting endosome (ASE) and next common recycling endosome (CRE), from where they can be directed into vesicles either to the basolateral membrane or multivesicular bodies (MVBs). When an MVB fuses with lysosomes, endolysosome (EL) is formed; however, disruption of this process may promote the fusion of virion-containing MVB with the cytoplasmic membrane. *Right*: the presence of an LC3-interacting region motif in the cytoplasmic tail of ACE2 may suggest interaction of vesicles with virions with the phagophore and their accumulation in the autophagosome. Factors that prevent the fusion of autophagosomes with lysosomes create conditions for their fusion with the cytoplasmic membrane.

It is likely that intestinal epithelium and M-cells can carry out virion transcytosis through common recycling endosomes (**Figure 1**, left) after binding of SARS-CoV-2 to ACE2 and cleavage of the receptor with cellular proteases such as TMPRSS2 (34, 35). At the same time, the cell entry mechanism of coronaviruses suggests that after common recycling endosomes, SARS-CoV-2 is more likely to end up in the multivesicular bodies/late endosomes, and after fusion with the lysosome, cathepsins trigger fusion of the virus envelope with the endolysosome membrane and cytoplasmic release of the virus genetic material (80). Although a number of randomized controlled trials have shown that hydroxychloroquine is not an effective drug for post-exposure prophylaxis (81) or treatment (82) of COVID-19, several studies are still underway to prevent and treat COVID-19 with chloroquine and hydroxychloroquine (42). It is assumed that these drugs stabilize lysosomal membranes and prevent acidification of the late endosome environment (42, 83). This may increase the likelihood that multivesicular bodies merge with the basolateral membrane and that the luminal contents, including viruses, will be thrown into the extracellular space (84) (**Figure 1**, left).

There is also a potential variant of viral transcytosis when exocytosed viral particles are enclosed in membrane vesicles. One possible mechanism for this phenomenon is involvement of the autophagy process (**Figure 1**, right). It was already mentioned that ACE2’s cytoplasmic tail has an LC3-interacting region motif that can determine interaction with the phagophore and autophagosome (57, 59). Inhibition of the autophagosome-lysosome fusion can create conditions that promote fusion of the autophagosome outer membrane with the cytoplasmic membrane and subsequent release of the inner membrane and its contents into the extracellular space (85). This process can be enhanced by inhibiting lysosomal degradation with inhibitors of lysosomal acidification (85) or by other pathological processes that can promote exocytosis of the inner membrane of the autophagosome. In addition, the LC3 protein that plays a central role in autophagy was localized in exosomes produced by multivesicular bodies (86). This raises the possibility of fusion between autophagosomes and multivesicular bodies with release of the inner membrane structures of autophagosomes in the form of extracellular vesicles.

The possibility of including virions into autophagosomes and subsequent release from cells in membrane vesicles has been shown for non-enveloped picornaviruses such as poliovirus, rhinoviruses, and Coxsackie type B virus (87–89). Some herpesviruses such as varicella-zoster and Epstein-Barr are able to use the autophagosome membranes and prevent their fusion with lysosomes, enabling them to form envelopes when released from the cell (90–92). Influenza A and parainfluenza viruses can block the fusion of autophagosomes with lysosomes, redirecting the autophagosome membranes to the viral budding site of the cytoplasmic membrane (93–95). RNA-containing flaviviruses such as hepatitis C, Dengue, and Chikungunya viruses can use autophagosome membranes for exocytosis from cells using multivesicular bodies (96–99). The mouse hepatitis virus (MHV) from the coronavirus family stimulates the formation of double-membrane structures similar to autophagosomes, and its replication apparatus colocalizes with these structures (100). The SARS-CoV replication apparatus also colocalizes with autophagosome membranes (101). SARS-CoV and MERS-CoV can stimulate autophagosome formation but at the same time block their fusion with lysosomes (102, 103). SARS-CoV-2 is also able to limit the fusion of autophagosomes with lysosomes (104). As for the other viruses listed above, this may contribute to the release of SARS-CoV-2 particles or their elements from the cell in autophagosome membranes (**Figure 1**, right).

In human hepatoma cell culture infected with hepatitis C virus, extracellular vesicles contain virions and can infect healthy cells (105). Hepatitis A virus can also be secreted from human cells in extracellular vesicles. In Caco-2 cells, this form of the virus is mainly secreted from the apical membrane (<1% from the basolateral membrane); in HepG2-N6 cells, 36% and 64% of viruses are shed from the apical and basolateral sides, respectively (106). The possibility of transcytosis through human cells without infecting them was shown for HIV, while the viral particles or parts of them got into multivesicular bodies and were released in extracellular vesicles capable of infecting surrounding healthy cells (107–109). Apparently, preventing the fusion of autophagosomes and multivesicular bodies with lysosomes and the release of viruses from the cell in the membrane envelopes most often requires infection of the host cell. However, it is possible that during autophagy, endosomes or transport membrane vesicles with coronavirus particles can be captured in forming autophagosomes, and stabilization of lysosomes and autophagosomes will facilitate the release of the autophagosome contents, including viruses, to the extracellular space without infecting the cell itself (**Figure 1**, right). It is assumed that viral use of extracellular vesicles allows them to avoid the body’s immune system and contributes to more effective infection because these vesicles are absorbed by other cells without having to bind to specific virus receptors (110). It is possible that coronaviruses including SARS-CoV-2 can undergo transcytosis across epithelial barriers while in extracellular vesicles.

In addition to endocytosis of opsonized viral particles into immune cells, viral endocytosis and transcytosis across tissue barriers by Fc receptor-expressing cells are also possible. Transcytosis across tissue barriers has been shown for enveloped viruses. In low pH conditions, HIV coated with specific antibodies can penetrate the epithelial layer of the genital tract mucosa *via* the FcRn molecule, the Fc receptor of IgG (111). Similarly, cytomegalovirus can be transported across the syncytiotrophoblast of the placental barrier. With a high titer of neutralizing antibodies, no infection develops in cytotrophoblast cells after transcytosis; however, medium and low levels of neutralizing antibodies, as well as antibodies with low neutralizing activity, can lead to infection (112). FcRn has been shown to be capable of transcytosis of antibody-antigen complexes across the intestinal barrier *in vitro* and *in vivo* (113). Interestingly, ADE in HIV infection may be associated with simultaneous interaction of the opsonized virus with FcγR and the CD4 molecule, which is the cell target for HIV infection (114). In this regard, it can be assumed that SARS-CoV-2 may also undergo endocytosis and transcytosis due to antibody-dependent interaction with Ig receptors, but this does not preclude the possibility of simultaneous interaction with ACE2.

Radioactively labeled ^125^I-S1 protein is transported across the mouse blood–brain barrier, and this process was enhanced by wheatgerm agglutinin suggesting adsorptive mechanism of S1 protein transcytosis connected with cell-surface glycoproteins that contain sialic acid or N-acetylglucosamine (115). Authors speculate that if the viral binding protein crosses the blood-brain barrier, it is likely that protein enables the virus to cross the blood-brain barrier as well (115). This assumption is consistent with the detection of SARS-CoV-2 in the cerebrospinal fluid, although neuronal retrograde dissemination is also possible route of the nervous system invasion (116).

Syncytiotrophoblast of placental barrier can be infected by SARS-CoV-2 which was confirmed by molecular and immunohistochemical assays and electron microscopy. Authors observed high maternal serum titers of anti-S-protein antibodies and suggested that potential mechanism of placental invasion in this case would be antibody-dependent transcytosis mediated by FcRn (117). Another report also confirmed placental syncytiotrophoblast SARS-CoV-2 invasion by electron microscopy (118). Positive anti-SARS-CoV-2 IgM in blood of newborns delivered by cesarian from mothers infected with SARS-CoV-2 suggest in utero exposure to the SARS-CoV-2 virus (119, 120). In addition to infection of the placenta, the mechanism of vertical virus transmission can be due to the transcytosis of opsonized or free viruses or the transfer of viruses by infected blood cells, but more clinical and experimental studies are required to confirm these mechanisms (121).

Obviously, the fate of antibody-antigen complexes on the surface of epithelial barriers, including opsonized viral particles, depends on the properties of antibodies and the density and transport mechanisms of the corresponding antibody receptors in the target cell. All Ig types including IgA, IgD, IgE, IgG, and IgM are found in mucous membrane secretions, but their ratio, structure, and biological properties differ from those in blood plasma. The IgA, IgM, and IgG classes play the main role in antiviral immunity (122).

## Coronavirus Paracellular Trafficking

Another potential mechanism for viruses to cross tissue barriers is paracellular trafficking. HIV-1 infection compromises the blood-brain barrier integrity by decreasing the expression of tight junction proteins, extracellular matrix disruption by metallopeptidases, and inflammation (123). Rotavirus infection disrupts tight junctions in the intestinal epithelium and Caco-2 monolayer (124). The tight junction proteins JAM-A, occludin, and ZO-1 play an important role during rotavirus entry into MA104 cells (125). Hepatitis C virus binds to occludin and claudin-1 as tight junction-associated coreceptors to enter the cell (126). Human papilloma virus deregulates β-catenin and ZO-1 expression in adherent and tight junctions respectively potentially disrupting the cellular barrier (127). Rotaviruses and reoviruses disrupt intercellular junctions to access their receptors on the basolateral membrane (128, 129). West Nile and dengue viruses modify the structure of tight junctions to enter the bloodstream (130, 131).

PDZ domains have different cellular functions including protein transport and metabolism, cell-cell communication, and cell polarization (132). PDZ-binding motifs were discovered in proteins of different viruses, such as human T-lymphotropic virus type 1, human papilloma virus, human adenovirus type 9, hepatitis B and C viruses, Kaposi sarcoma herpesvirus, human immunodeficiency virus, providing interaction with PDZ-domain-containing proteins and enhancing viral replication, dissemination, and immune system evasion (132–134). SARS-CoV and SARS-CoV-2 E-proteins also have PDZ-binding motif (135). It is proposed that the interaction of SARS-CoV-1 E-protein with PALS1, the protein of an apical cell polarity complex, recruits PALS1 to the site of virus assembly, possibly disrupting intercellular tight junctions and increasing epithelial permeability for virions (136). E-proteins also interact with tight junction protein ZO-1 (137), adhesion junction protein syntetin (138), and other cell junction proteins (139). The redistribution of these molecules might contribute to cell junction impairment in lung epithelium and vessels during SARS-CoV-2 infection (135). The SARS-CoV-2 S-protein alters blood-brain barrier function both in 2D static and 3D microfluidic models (140). SARS-CoV-2 infection of *in vitro* cultured polarized human airway epithelium leads to dispersed ZO-1 expression without clear tight junctions (141).

## Using Tissue Barrier Models to Study Coronavirus Transcytosis

Due to the economic and social effects of COVID-19, it is highly relevant to study the mechanisms of infection and search for new effective drugs capable of fighting coronaviruses. Since animal studies are lengthy and often do not yield results when specific pathogens are studied or when the studied drugs use specific features of human biology, animal-free approaches including *in vitro* studies using microfluidic technologies and various cell models based on organoids consisting of human cell lines (142–144) are increasingly important.

Explant cultures can be used as organ models for viral infection research because they reproduce natural morphology and microenvironment of organs. However, such models have low availability due to insufficient donor material, short viability, and low reproducibility of research results (145).

The human adenocarcinoma cell line Caco-2 is successfully used as a model of the human intestinal barrier. During long-term cultivation, Caco-2 cells polarize, form microvilli, tight junctions, extracellular matrix, and begin to express specific enterocyte markers (146–148). Models based on Caco-2 cells using microfluidic devices that create medium flow make the model even closer to the physiological conditions of human intestine (149–151). The lung-on-chip model in a microfluidic device can be used to study influenza A virus cell entry, replication, virulence of different strains, cytokine production by host cells, and circulating immune cell recruitment (152). The same model was used to study the effect of seven clinically approved drugs on the entry of virus-like particles with SARS-CoV-2 S-protein, and differences in drug effects were noted compared to the cell model outside the microfluidic chip (152). It can be assumed that the gut-on-a-chip model based on Caco-2 cells will also differ from cells in a static culture medium.

In an intestinal model based on Caco-2 cells, SARS-CoV-2 infection depends on ACE2 and TMPRSS2 (6). A study of 13 human cell lines showed that only Caco-2 had sufficient ACE2 expression and was susceptible to SARS-CoV infection *in vitro* (153). Among seven other human colorectal adenocarcinoma cell lines (DLD-1, HCT-116, HT-29, LoVo, LS-180, SW-480, and SW-620) only LoVo cells were susceptible to SARS-CoV infection, but this did not correlate with *ACE2* gene expression, which was higher in LS-180 and SW-620 by about 7 and 50 times, respectively (154). In another study, among colorectal adenocarcinoma cell lines Caco-2, CL-14, HT-29, SW-480, DLD-1, and HCT-15 only Caco-2 and CL-14 were susceptible to SARS-CoV (155).

According to a study of the proteome of filter-grown Caco-2 cells (156), there are ~1.7×10^5^ copies of the p51 FcRn subunit and ~1.5×10^6^ copies of β2-microglobulin in each Caco-2 cell. Considering that the same study reported ~2.0×10^4^ ACE2 molecules and ~2.7×10^4^ NRP2 molecules per Caco-2 cell, while NRP1 was not detected, cell entry of opsonized virion through the antibody complex with FcRn may be highly probable. It was previously shown that infection with the porcine transmissible gastroenteritis virus (TGEV) belonging to the coronavirus family can cause an increase of FcRn expression in the normal porcine intestinal cell line IPEC-J2 by activating NF-κB signaling through membrane receptors of the Toll-like receptor family and increasing pro-inflammatory cytokine levels (157, 158). Conversely, infection with the CHN-JS-2017 strain of porcine deltacoronavirus PDCoV in piglets was associated with NF-κB pathway suppression and decreased FcRn and pIgR expression in the intestinal epithelium, which may enable the virus to avoid secreted neutralizing antibodies (159). Thus, SARS-CoV and SARS-CoV-2 infection can both theoretically increase FcRn and pIgR expression and antibody-mediated virus transcytosis across the intestinal barrier.

We previously published the results of a transcriptome study using Affymetrix Human Gene 1.0 ST Arrays (GSE81867) for Caco-2 cells grown either on Transwell culture inserts in static medium or on a microfluidic chip with culture medium circulation (150, 151). Further transcriptome analyses revealed high *FCGRT* gene expression in differentiated Caco-2 for both conditions. At the same time, the expressions of *ACE2*, *PIGR*, *NRP1*, and *NRP2* genes in these cells were significantly lower. This is consistent with the proteome data of Caco-2 cells, in that ACE2 and NRP2 levels were significantly lower than FCGRT level, while pIgR and NRP1 were not detected (156).

Song et al. investigated the intracellular transport of micelles with a size of 30–40 nm, coated with FcBP, the peptide ligand of FcRn (160). These micelles may mimic SARS-CoV-2 virions covered with immunoglobulins because both of them have Fc regions on their surface, therefore it is reasonable to assume that the intracellular transport pathways of FcBP-decorated micelles may be a model of opsonized coronavirus particle transport. The analysis of micelle transport pathways in Caco-2 cells revealed their inclusion in the pathways of recirculation and transcytosis through common recycling endosomes and the endoplasmic reticulum, but not the Golgi complex (160). Macropinocytosis inhibitors 5-(N-ethyl-N-isopropyl)-amiloride (EIPA) and cytochalasin D, as well as the tyrosine-specific protein kinase inhibitor genistein had no effect on FcRn-mediated transport. Dynasore, which inhibits dynamin-dependent clathrin vesicle formation during endocytosis and disrupts lipid raft structure, reduced FcRn-mediated micelle uptake by 20%. Hypertonic sucrose solution that prevents clathrin vesicle formation also significantly suppressed micelle uptake, while chlorpromazine, which inhibits AP2 subunit assembly during clathrin-mediated endocytosis, had practically no effect on FcRn-mediated uptake. When analyzed using transport inhibitors associated with caveolae and lipid rafts, the results revealed that filipin slightly increased FcRn-mediated micelle uptake, nystatin slightly decreased it, and methyl-β-cyclodextrin significantly inhibited FcRn-mediated uptake. Thus, it was concluded that FcRn-mediated endocytosis can be simultaneously associated with both clathrin-coated vesicles and caveolae and lipid rafts (160).

Analysis of the transport pathways of FcBP-decorated micelles revealed that they are only weakly retained in apical sorting endosomes, actively pass through common recycling endosomes during transcytosis toward the basolateral membrane, are partially transported to the apical recycling endosomes and endoplasmic reticulum to return to the apical membrane, but are not transported through the Golgi complex and practically do not enter basal recycling endosomes as this compartment is associated with basolateral membrane endocytosis. At a relatively low FcRn ligand density, micelles were successfully avoided late endosomes and did not fuse with lysosomes; however, this pathway significantly increased at a high ligand density (160).

**Figure 2:**
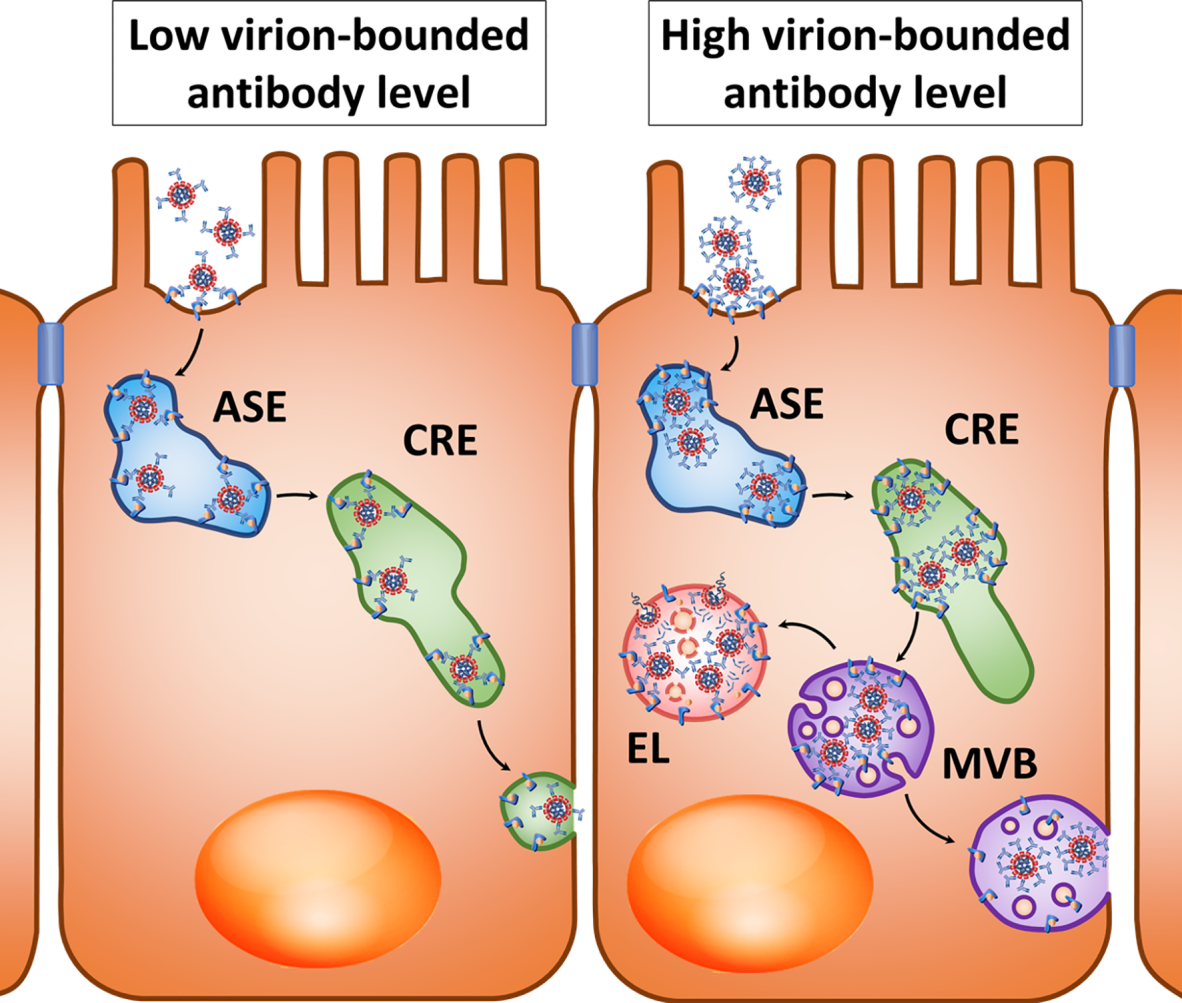
Hypothetical pathways for transcytosis of the opsonized SARS-CoV-2 after interaction with the FcRn, Fc-region receptor in a polarized cell. *Left*: At a relatively low virion-bounded antibody level, coronavirus transcytosis is possible through the tubules of apical sorting endosome (ASE) and common recycling endosome (CRE). *Right*: With a high density of virion-bounded antibodies or virion agglomerate formation, sorting in CRE may be changed, resulting in virions entering the multivesicular bodies (MVBs) that can either fuse with lysosomes to form endolysosome (EL), or fuse with the cytoplasmic membrane when lysosome fusion is suppressed.

The described transport mechanisms suggest that Ig-coated SARS-CoV-2 may undergo transcytosis across the intestinal barrier and other tissue barriers whose cells express FcRn, and with a high level of antibodies increased transport to late endosomes and fusion with lysosomes can be expected. However, it has already been mentioned that the use of lysosome lumen acidification inhibitors can lead to multivesicular bodies merging with the basolateral membrane to promote viral transcytosis (**Figure 2**, right).

The polarity of transcytosis involving FcRn deserves special consideration in light of the potential for infection with coronaviruses involving this receptor. It was shown that Caco-2 cells transfected with the murine *Ceacam1* gene, which is a receptor of MHV, were successfully infected with this coronavirus, while the release of new viral particles occurred on the basolateral surface (161).

Within endothelial cells, FcRn is primarily localized in early endosomes positive for Rab5 and EEA1 and in recycling endosomes positive for Rab4 and Rab11 (162, 163). Ig molecules are believed to penetrate into endothelial cells mainly due to pinocytosis and bind to FcRn under conditions of low intracellular pH, and they then undergo further sorting (40). During antigen opsonization involving large amounts of antibodies and “cross-linking” of several FcRn molecules by polymerized IgG, immune complexes in the endothelium are predominantly directed to lysosomes, while FcRn associated with monomeric Ig molecules allows the targeting of immune complexes in recycling endosomes (164). This is consistent with the previously mentioned results obtained in polarized Caco-2 cells, in which the high density of the FcRn ligand on the surface of artificial micelles induced a decrease in transcytosis and increased sorting of these micelles into lysosomes compared with low ligand density (160). This may be explained by the fact that the high density of complexes of ligands and antibodies prevents penetration of opsonized particles into the narrow sorting channels of endosomes (165). Perhaps penetration into narrow sorting channels is hindered by the size of “cross-linked” immune complexes, as has been shown for sorting different sizes of cargoes in macrophages (166). Another possible cause is steric mismatch between cross-linked FcRn-IgG complexes to the large curvature of endosome-sorting tubules (167) (**Figure 2**, right).

The analysis of FcRn transcytosis in polarized cells was largely performed in MDCK II canine renal epithelial cells. The transcytosis mechanisms in these cells differed from the receptor recirculation mechanism: FcRn-mediated transcytosis occurred in both directions and depended on myosin Vb and GTPase Rab25, while FcRn recirculation depended on Rab11a and was only carried out in the direction of the basolateral membrane (168). Transcytosis was also dependent on the binding of calmodulin to the cytoplasmic tail of FcRn and required endosome medium acidification (40). When MDCK II cells were transfected with human FcRn, it predominantly localized in apical intracellular vesicular structures and the basolateral membrane, while removal of the cytoplasmic tail of FcRn containing the dileucine and tryptophan motifs of endocytosis led to receptor redistribution into the apical membrane. Endocytosis of full-length FcRn was carried out equally from the apical and basolateral sides. The authors concluded that the observed intracellular distribution of FcRn is associated with the predominant direction of transcytosis from the apical to basolateral cell surface, while maintaining the possibility of transcytosis in the opposite direction (169).

In a model of intestinal epithelium based on polarized cells of colorectal adenocarcinoma T84, FcRn was mainly intracellular and on the apical membrane of cells, with transcytosis involving FcRn proceeding in both directions (170). In human small intestine epithelial cells, the receptor was also localized predominantly apically (171). A study of polarized Caco-2 cells suggested that Ig endocytosis from the apical membrane may occur mainly due to pinocytosis rather than binding to the receptor, and binding to FcRn occurs in endosomes already inside the cell at a low pH (172). Others demonstrated that FcRn can transport monomeric IgG from the basolateral to apical membrane of intestinal cells and then transport Ig complexes with antigens in the opposite direction to present antigens as immune complexes to dendritic cells of the mucous (40). The immune complexes of antibodies and antigens could also be transported from the basolateral to apical membrane (113). These results suggest the possibility of SARS-CoV-2 transcytosis across epithelial barriers due to FcRn.

Models based on primary intestinal epithelial cells, small intestine explants, and intestinal organoids are susceptible to MERS-CoV infection and able to support sustainable virus replication. Caco-2 cells grown on Transwell culture inserts were more susceptible to MERS-CoV upon inoculation of the virus from the apical side than from the basolateral side, while inoculation of the virus into the stomach of a transgenic mouse expressing human DPP4 led to intestinal infection, spread of infection to the respiratory tract, and death (173).

Another promising tool to study SARS-CoV-2 infection is organoids, self-organized three-dimensional tissue or cell cultures representing miniaturized and simplified models of organs. Intestinal organoids derived from horseshoe bats and humans that can recapitulate intestinal mucosa were susceptible to SARS-CoV-2 infection and sustain viral replication (174). Human small intestinal organoids were readily infected by SARS-CoV and SARS-CoV-2 producing infectious viral particles (8). Remdesivir effectively inhibited SARS-CoV-2 infection in human intestinal organoids derived from pluripotent stem cells (175). Lung and intestine organoid models were used for high-throughput screening to identify SARS-CoV-2 entry inhibitors (176). Human bronchial organoids consisting of basal, club, ciliated, and goblet cells were susceptible to SARS-CoV-2 infection and allow to study camostat inhibitory effect on virus entry (177). Cell and organoid derivatives from human pluripotent stem cells were incubated with Spike-enabled pseudo-entry virus to study SARS-CoV-2 tissue tropism. Pancreatic endocrine cells, liver organoids, cardiomyocytes, dopaminergic neurons, adult primary human islets, adult hepatocyte and cholangiocyte organoids were highly permissive to SARS-CoV-2 infection (178). Brain organoids provide a useful model for investigating SARS-CoV-2 entry into the human brain and elucidating the susceptibility of the brain to SARS-CoV-2 (179–181). Human induced pluripotent stem cell-derived BrainSphere model was used to show SARS-CoV-2 infection of neural cells expressing ACE2 but not TMPRSS2, however authors do not model how viruses enter brain parenchyma (182). Induced pluripotent stem cells-derived human neural progenitor cells, neurospheres, and brain organoids were permissive to SARS-CoV-2 and showed signs of productive infection (183). SARS-CoV-2 can directly infect engineered human blood vessel organoids and human kidney organoids, which can be inhibited by soluble human ACE2 (184). It should be concluded that organoids make it possible to study the tropism of coronaviruses and to search for potential drugs against SAS-CoV-2; however, using these models, it is difficult to reproduce the penetration of viruses through barrier tissues.

The possibilities of using microfluidic kidney-on-a-chip (185) and lung-on-a-chip (186) models for studying acute kidney injury and lung infection, respectively, have been proposed but not used to study SARS-CoV-2. In the microfluidic model of the human blood-brain barrier, administration of different SARS-CoV-2 S-protein subunits induced a pro-inflammatory response and impaired the model’s barrier function (140). Models of human organs, including microfluidic platforms, have been used to study various viral infections (187, 188), which can be adapted and optimized to investigate coronavirus infections.

## Potential Clinical Targeting of SARS-CoV-2 Barrier Penetration

The possibility of SARS-CoV-2 hijacking the endocytosis and transcytosis mechanisms in barrier tissues makes it relevant to search for new inhibitors and modulators of these processes. Different endocytic pathways can be targeted *in vitro* by known pharmacological blockers such as amiloride for macropinocytosis, methyl-β-cyclodextrin for dynamin‐independent endocytosis, nystatin for caveolae-mediated endocytosis, dynasore and pitstop for clathrin‐dependent endocytosis (189). One of the main approaches for developing drugs against SARS-CoV-2 is the repurposing of previously approved drugs. As was mentioned above, there are several studies of chloroquine and hydroxychloroquine as SARS-CoV-2 entry blockers through endosomal acidification, although their effectiveness is controversial. From previously approved drugs chlorpromazine, promethazine, and vinblastine can regulate clathrin-dependent endocytosis (190–192), fluvoxamine and sertraline affect dynamin-dependent endocytosis (193, 194), nystatin is used to block caveolae (190), and macropinocytosis can be targeted by amiloride, flubendazole, imipramine, itraconazole, terfenadine, and vinblastine (195, 196).

The ClinicalTrials.gov search revealed two clinical trials of chlorpromazine, seven trials of fluvoxamine, and two studies of itraconazole for COVID-19 treatment, details are summarized in **Table 1**. Other mentioned above drugs are not studied for COVID-19 treatment in clinical trials yet. Sertraline is studied only as a part of the usual treatment of post-traumatic stress disorder in COVID-19 survivors comparing to Basic Body Awareness Therapy (NCT04396314). One trial is aimed to study the pharmacokinetics, pharmacodynamics, and safety profile of understudied drugs including sertraline administered to children with COVID-19 and other conditions per standard of care (NCT04278404). There are no clinical trials studying amiloride as primary treatment for COVID-19, but it is one of many drugs for hypertension treatment in patients with COVID-19 in the trial NCT04467931.

**Table 1:** Clinical trials of potential endocytosis and transcytosis inhibitors and modulators.

	Drug	Regulated endocytic pathway	Clinical trials related to COVID-19	Phase	Interventions	Status	ClinicalTrials.gov Identifier
1	Chlorpromazine	Clathrin	Repurposing of Chlorpromazine in Covid-19 Treatment	Phase 3	Drug: Chlorpromazine Combination Product: Standard of Care	Not yet recruiting	NCT04366739
2	Chlorpromazine	Clathrin	Administration of Chlorpromazine as a Treatment for COVID-19	Phase 2 Phase 3	Drug: Chlorpromazine	Not yet recruiting	NCT04354805
3	Fluvoxamine	Dynamin	Fluvoxamine Administration in Moderate SARS-CoV-2 (COVID-19) Infected Patients	Phase 2	Drug: Placebo Drug: Fluvoxamine	Recruiting	NCT04718480
4	Fluvoxamine	Dynamin	A Double-blind, Placebo-controlled Clinical Trial of Fluvoxamine for Symptomatic Individuals With COVID-19 Infection (STOP COVID)	Phase 2	Drug: Fluvoxamine Drug: Placebo	Completed: patients treated with fluvoxamine, compared with placebo, had a lower likelihood of clinical deterioration over 15 days (197)	NCT04342663
5	Fluvoxamine	Dynamin	Fluvoxamine for Early Treatment of Covid-19 (Stop Covid 2)	Phase 3	Drug: Fluvoxamine Drug: Placebo	Active, not recruiting	NCT04668950
6	Fluvoxamine	Dynamin	Fluvoxamine for Adults With Mild to Moderate COVID-19	Phase 2	Drug: Fluvoxamine Drug: Placebo	Suspended (Closure of main community treatment center)	NCT04711863
7	Fluvoxamine	Dynamin	Outpatient Treatment of SARS-CoV-2 With Ivermectin, Fluvoxamine, and Metformin (COVID-19)	Phase 2 Phase 3	Drug: Metformin Drug: Placebo Drug: Fluvoxamine Drug: Ivermectin	Recruiting	NCT04510194
8	Fluvoxamine	Dynamin	Repurposed Approved and Under Development Therapies for Patients With Early-Onset COVID-19 and Mild Symptoms	Phase 3	Drug: Fluvoxamine Drug: Doxazosin Drug: Ivermectin Drug: Placebo Drug: Peginterferon Lambda-1a Drug: Peginterferon Beta-1A	Recruiting	NCT04727424
9	Fluvoxamine	Dynamin	ACTIV-6: COVID-19 Study of Repurposed Medications	Phase 3	Drug: Ivermectin Drug: Fluvoxamine Drug: Fluticasone Other: Placebo	Recruiting	NCT04885530
10	Itraconazole	Macropinocytosis	Efficacy and Safety of Drug Combination Therapy of Isotretinoin and Some Antifungal Drugs as A Potential Aerosol Therapy for COVID-19: An Innovative Therapeutic Approach COVID-19 (Isotretinoin)	Phase 2	Drug: Aerosolized Isotretinoin plus Aerosolized Itraconazole Drug: Aerosolized Isotretinoin	Not yet recruiting	NCT04577378
11	Itraconazole	Macropinocytosis	Drug-Drug Interaction Study Assessing Effect of Itraconazole on PF-07321332/Ritonavir in Healthy Participants	Phase 1	Drug: PF-07321332/ritonavir Drug: Itraconazole	Recruiting	NCT04962022

As was mentioned above, PDZ-binding motifs might play role in different viral infections. The human papilloma virus PDZ-domain-containing protein E6 was targeted in different studies to prevent infection and oncogenic transformation of cervical epithelial cells. Therapeutic targeting of E6 started with peptide-based inhibition but these peptides had low affinity and weak activity in cancer cell lines (198). High-affinity bivalent binders of the E6 protein were designed by linking of its cellular PDZ-domain-containing targets, but their activity was studied only *in vitro* (199, 200). The complex of E6 PDZ-binding motif and PDZ domain was used to screen a commercial compound database revealing two potential regulators but experimental validation was not presented (201). *In vitro* study revealed the potential of fusicoccin to prevent interaction of the E6 PDZ-binding motif with 14-3-3 protein isoforms without *in vivo* validation (202).

There are also studies of drugs potentially preventing cell SARS-CoV-2 entry by TMPRSS2 protease regulation. TMPRSS2 inhibitors include camostat mesylate, nafamostat mesylate, and antiandrogens (203). There are 25 trials of camostat mesylate, 7 trials of nafamostat mesylate, 2 trials of enzalutamide, 6 trials of proxalutamide, 1 trial of dutasteride, 2 trials of enzalutamide, and 3 trials of bicalutamide on ClinicalTrials.gov. Another study revealed a novel potential SARS-CoV-2 entry route through host cell receptor CD147 and showed, that the anti-CD147 antibody, meplazumab, inhibits SARS-CoV-2 amplification *in vitro* (204). The Phase 1/Phase 2 clinical trial of meplazumab for treatment of COVID-19 is completed (NCT04275245) and Phase 2/Phase 3 trial is recruiting patients (NCT04586153). These trials were not the subject of this review as well as studies of angiotensin receptor blockers, calmodulin antagonists, selective oestrogen receptor modifiers, and recombinant soluble ACE2 as ACE2 modulators (203).

Currently, the expression of ACE2 is accounted critical for SARS-CoV-2 binding and entry. Nonetheless, it is important to identify new mechanisms that the virus may employ to its advantage for a complete pathogenic cycle, including endocytosis and transcytosis hijacking by SARS-CoV-2. As we mentioned above, several trials are studying potential inhibitors and modulators of endocytosis and transcytosis, but their results mostly have not been published yet.

## Conclusion

Several studies suggest that the gastrointestinal tract may be an entry portal and reservoir for SARS-CoV-2 infection. Native SARS-CoV-2 virions have sparsely located highly mobile S-proteins on their surface, which enables active interaction with both specific target cell receptors and immunoglobulins. Native SARS-CoV-2 virions undergo endocytosis when S-proteins interact with ACE2 or NRP1 with the assistance of cellular proteases. Opsonized SARS-CoV-2 virions are coated with antibodies that mask viral proteins, but the virus can still bind the cell surface and be endocytosed due to the presence of receptors for antibody Fc regions. This normally promotes absorption of virions by immune cells for antigen presentation and pathogen neutralization; however, the phenomenon of ADE occurs when the concentration of antibodies is too low or they do not have sufficient specificity and neutralizing activity to prevent intracellular viral replication. ADE has been experimentally shown for the SARS-CoV, MERS-CoV, and SARS-CoV-2 coronaviruses, but the clinical feasibility of ADE in these infections remains unclear.

Epithelial cells of barrier tissues in the intestine, lungs, kidneys, and placental trophoblast express the IgG FcRn receptor, which mediates transcytosis of antigen-antibody complexes for immune cell presentation. At the same time, complexes of antigens with polymeric immunoglobulins (IgA and IgM) and their receptor pIgR do not seem to be effectively transported across the epithelial barrier towards the basolateral membrane. However, IgG-dependent transcytosis of viral particles can allow enveloped viruses (e.g., HIV and cytomegalovirus) to penetrate barrier tissues. In this regard, the possibility of coronavirus penetration through the epithelial barrier of the intestine and other organs by an antibody-dependent mechanism cannot be denied. This possibility is supported by the high level of FcRn receptor expression in the gastrointestinal tract and lungs. At the same time, the Caco-2 cell line expressing ACE2 and FcRn and some other models in static culture medium and microfluidic devices can be used as suitable intestinal barrier models for studying coronavirus transcytosis. Another possible pathway for viruses to penetrate tissue barriers is paracellular trafficking *via* disruption or modulation of cell-cell junctions. SARS-CoV-2 E-protein has a PDZ-binding motif which could bind cell junction proteins and cause their redistribution from junctions to the site of virus assembly, leading to barrier impairment.

The repurposing of previously approved drugs that can target different endocytic pathways allows studying potential endocytosis and transcytosis inhibitors and modulators to fight COVID-19. There are several registered clinical trials of chlorpromazine, fluvoxamine, and itraconazole, but they lack published results, and more trials are needed to find new treatment modalities for COVID-19.

## Future Directions

An increase in the number of people recovered from COVID-19 and the use of vaccines and convalescent plasma treatment suggests that we will increasingly encounter questions of the interaction of coronavirus with human antibodies. The emergence of new strains of coronavirus may potentially lead to the problem that existing antibodies will not have full neutralizing activity, which underlies such a phenomenon as antibody-dependent enhancement of infection. A decrease in antibody titers in recovered people may also lead to this phenomenon. In this regard, one of the necessary directions of research is the observation of cases of SARS-CoV-2 re-infection in order to find out the specificity of antibodies to newly emerging strains. Also, special attention should be paid to the cases and mechanisms of overcoming tissue barriers within the body by the coronavirus, including the blood-brain and placental barriers. These clinical studies should be accompanied by *in vitro* studies, which makes it urgent to develop relevant models of various aspects of the coronavirus interaction with the human body. Experiments will help confirm or disprove the ability of coronavirus to overcome tissue barriers by transcytosis, including antibody-mediated transcytosis. The repurposing of previously approved drugs to target coronavirus endocytosis and transcytosis may be useful for fast COVID-19 treatment development without preclinical testing.

## Author Contributions

AT conceptualized and reviewed the work. SN worked on analysis of the bioinformatics data presented. EK worked on curation, analysis and visualization of the data presented, prepared the original draft, edited and reviewed it. All authors contributed to the article and approved the submitted version.

## Funding

The reported study was funded by RFBR, project number 20-04-60399 (AG, EK); Basic Research program at HSE University and funded by the Russian Academic Excellence Project ‘5-100’ (SN).

## Conflict of Interest

The authors declare that the research was conducted in the absence of any commercial or financial relationships that could be construed as a potential conflict of interest.

## Publisher’s Note

All claims expressed in this article are solely those of the authors and do not necessarily represent those of their affiliated organizations, or those of the publisher, the editors and the reviewers. Any product that may be evaluated in this article, or claim that may be made by its manufacturer, is not guaranteed or endorsed by the publisher.
